# From pill to pain: Alendronate‐induced esophageal injury—A case report and review

**DOI:** 10.1002/jgh3.13080

**Published:** 2024-06-02

**Authors:** Zhen Fan, Hayat Khizar, Jinjiao Lu, Anhua Wang, Tong Xun, Xiao Zhang, Hongfeng Zhao

**Affiliations:** ^1^ Department of Gastroenterology Hangzhou First People's Hospital Hangzhou China; ^2^ The Fourth School of Clinical Medicine Zhejiang Chinese Medical University Hangzhou China; ^3^ Department of Gastroenterology Wenling First People's Hospital Taizhou China; ^4^ Department of Pathology Hangzhou First People's Hospital Hangzhou China; ^5^ Department of Infection Control Hangzhou First People's Hospital Hangzhou China

**Keywords:** alendronate, case report, drug‐induced diseases, drug safety, esophagitis

## Abstract

**Background:**

Alendronate is used to treat Paget's bone disease, glucocorticoid‐induced osteoporosis, and postmenopausal osteoporosis because it suppresses osteoclast activity to stop bone resorption.

**Case report:**

We present an exceptional case of esophagitis caused by alendronate. Blood tests and other data were normal when the patient was taken to the hospital, but an endoscopic examination revealed significant esophageal redness, erosion, and ulceration, along with pseudomembrane. The patient was given medicine after receiving a diagnosis of alendronate pill‐induced esophagitis based on the pathological findings.

**Conclusion:**

This case report is a timely reminder of the importance of thorough pharmacovigilance, patient education, and smart therapeutic decision‐making in the context of alendronate use. To properly treat and prevent problems with the esophagus caused by alendronate, additional research is required.

## Graphical abstract

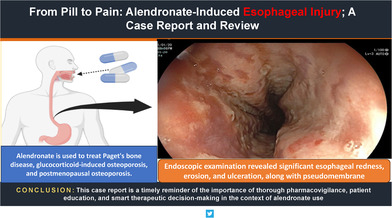

## Introduction

Alendronate sodium, with the chemical formula C4H12NNaO7P2, has a high affinity for intraosseous hydroxyapatite and inhibits osteoclast activity to prevent bone resorption. It has been widely used in the treatment of Paget's bone disease, glucocorticoid‐induced osteoporosis, and postmenopausal osteoporosis since its introduction in the United States in 1995,^1^, ^2^ and it is the first bisphosphonate drug approved by the Food and Drug Administration (FDA) for the treatment of postmenopausal osteoporosis, which can directly inhibit osteoclasts, promote osteoclast apoptosis, and then reduce bone turnover. It significantly increases bone density and reduces fracture incidence in postmenopausal osteoporosis patients. While it is effective in preventing and treating disease, there have been case reports abroad describing the damage to the upper digestive tract from the use of the drug, particularly erosive or ulcerative esophagitis.^3^, ^4^ In recent years, with the aging society, the application of alendronate sodium has become more and more extensive, and the resulting drug‐induced esophagitis (pill esophagitis) has increased, and some clinicians and endoscopists have insufficient understanding of the disease.^5^, ^6^

## Case report

A 62‐year‐old patient was admitted to our hospital with a chief complaint of “retrosternal pain and discomfort persisting for the past 3 days.” The patient reported the onset of continuous retrosternal pain and discomfort in the lower thoracic region, exacerbated by ingestion and accompanied by a sensation of choking. Notably, the patient denied symptoms such as acid belching, heartburn, hematemesis, a productive cough, or dyspnea. There was no history of exposure to corrosive agents or foreign body ingestion before symptom onset. Additionally, there was no recent history of significant weight loss. Relevant medical history included a thyroidectomy necessitating long‐term oral supplementation of levothyroxine sodium at a dose of 125 g once daily for the past 6 years, attributed to secondary hypothyroidism. One week before admission, the patient received a diagnosis of “osteoporosis” and was initiated on oral sodium alendronate therapy at a weekly dose. Physical examination revealed an elevated body temperature of 38.1°C (100.6 °F), while other clinical parameters were within normal limits.

## Laboratory tests and imaging findings

**Figure 1:**
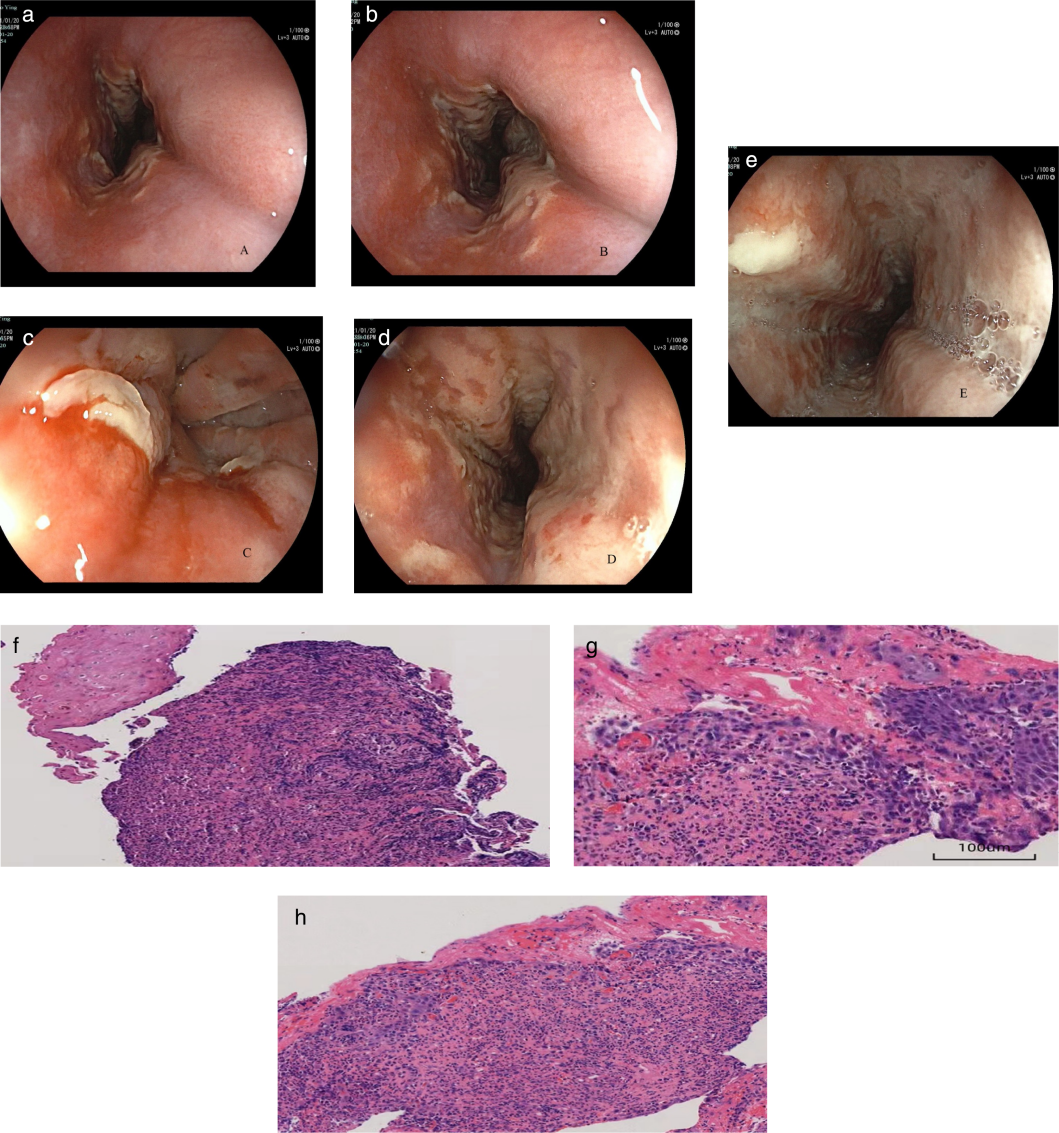
Endoscopic findings: Segmental extensive erosions, ulcers, and a loosely adherent pseudo‐membrane covered with dense inflammatory exudates (a–c). Esophageal segmental mucosa is widely red, eroded, with ulcer formation of different shades, circumferential, distributed along the esophageal wall in circular or kissing against each other, varying in size, partial ulcer fusion, clear ulcer boundaries, and the surface is covered with a prominent, dense, and thick white pseudomembranous exudate loosely adherent (a–e). Normal mucosa exists at both ends of the lesion mucosa of esophagus (a). Histopathologic findings (f–h): Inflammatory necrotic tissue with granulation tissue hyperplasia, a little squamous epithelium on the surface, and widening of the intercellular space with neutrophil and lymphocyte infiltration, consistent with ulcer changes (HE staining ×200 magnification).

1. Laboratory findings: Routine WBC(White blood cells) 13.1 × 109/L, neutrophil 77.7%, hemoglobin 146 g/L, CRP (C‐reactive protein) 25 mg/L. There were no abnormalities in blood biochemistry, myocardial enzymes, TnI (Troponin I), coagulation function, and tumor markers. Conventional ECG (Electrocardiogram) and chest CT (Computed tomography) scan showed no abnormalities.
2. Detailed Endoscopic Examination: The upper part of the esophagus appeared normal, with no evident abnormalities. Approximately 23–40 centimeters from the biting surface (incisors), noteworthy findings emerged. The inner lining of the esophagus displayed substantial redness, areas where the tissue had worn away (erosions), and the presence of ulcers with varying shades of color. These ulcers were accompanied by a white substance adhering unevenly to the surface, resembling a false membrane (pseudomembrane). These affected regions were apart from the surrounding healthy tissue, but within them, small patches of normal tissue were visible. While the diameter of the esophageal passage was slightly narrower. Biopsies were conducted at multiple sites within the affected segments for further analysis. Shifting the focus to the stomach and duodenal regions, no significant abnormalities were identified (Fig. 1a–e).
3. Histopathologic findings: Inflammatory necrotic tissue with granulation tissue hyperplasia, a little squamous epithelium on the surface, and widening of the intercellular space with neutrophil and lymphocyte infiltration, consistent with ulcer changes (Fig. 1f–h).

## Final diagnosis

1. Alendronate sodium‐related pill esophagitis^2^; osteoporosis^3^; post‐thyroidectomy.

## Treatment

After being admitted to the hospital, the patient received care that included fluid infusion support, fasting, pantoprazole for acid suppression, and ceftazidime for anti‐infection. The following day, a gastroscopy examination was performed, and it was discovered that the esophagus mucosal injury was segmental and circumferential, with distinct identified boundaries and healthy areas. Given this symptom, the medical history was reviewed again. It was discovered that the patient drank less than 50 mL of water when taking oral alendronate, and when oral alendronate sodium was taken for the first time, there were apparent indications of swallowing pain. The patient was prescribed a liquid diet, advised to stop taking alendronate sodium, and given an oral suspension of sucralfate to protect the esophageal mucosa after being given a precise diagnosis.

## Results and follow‐up

The patient showed an improvement to a normal body temperature on the fourth day after being admitted, and the regular blood test also returned to normal. The patient then successfully adjusted to a semi‐liquid dietary protocol, which led to the decision to discharge the patient from the hospital. The patient continued to be asymptomatic, with retrosternal pain, dysphagia, and dyspeptic burning during the evaluation conducted a month after being discharged from the hospital. Later, patients were stopped from using alendronate medication.

## Discussion

Alendronate‐induced esophagitis is a clinically rare drug‐induced esophagitis that can be seen in patients taking bisphosphonates for postmenopausal osteoporosis. A review of global post‐marketing surveillance data of alendronate estimated 475 000 patients worldwide, and 1213 reports of adverse effects had been received. A total of 199 patients had adverse effects related to the esophagus.^3^ On the other hand, multiple randomized, double‐blind controlled trials have shown that the overall incidence of upper gastrointestinal adverse reactions in patients treated with alendronate is similar to that of patients receiving placebo when taken as instructed.^7^, ^8^, ^9^ These results suggest that esophageal adverse reactions occur only to a small extent and are closely related to inappropriate dosage methods.

Drug‐induced esophageal injury is very common, and clinical symptoms of esophageal injury include painful swallowing, dysphagia, retrosternal chest pain, epigastric pain, and hematemesis.^10^ According to previous case reports, symptoms are usually detected within days to weeks after the start of medication, most cases recover within half a month, and some cases have been reported to be treated with oral drugs for up to 10 months, and eventually surgery due to esophageal stricture.^11^, ^12^ Previous studies show that patients who took alendronate sodium experienced severe swallowing difficulties. However, examinations after stopping the medication did not reveal any long‐term issues, including esophageal strictures, suggesting that the pain was only temporary and disappeared upon stopping the alendronate.^3^, ^13^ Therefore, the most important thing in the diagnosis and treatment of this disease is to find the real cause in time, avoid reinjury caused by drugs, and stop oral drugs if possible, so clinical doctors and digestive endoscopists should deepen their understanding of this kind of esophagitis.^14^

At present, the pathogenesis of alendronate‐induced esophagitis is mainly believed to be due to the high concentration of corrosive substances produced when the esophageal mucosa is exposed to drugs for a long time, causing chemical damage.^12^, ^15^ In the cases reported by Abraham et al. and Ribeiro et al.,^16^, ^17^ the vast majority of histopathology found granulation tissue and inflammatory exudate at the ulcer site, which is consistent with the results of this case. It was found that 60% of biopsy tissues contained polarizable foreign bodies with a clear, refractive, crystalline appearance and mixed with inflammatory exudates, suggesting a prolonged contact time between the drug and the mucosa. Ribeiro et al. further confirmed that the birefringence crystalline substance was indeed drug debris, and they also proposed that most of the esophageal mucosal damage caused by alendronate was caused by long‐term exposure to drugs. But in this case, the patient's history of onset drug use and the presence of segmental esophagitis surrounded by normal mucosa did not support this mechanism. Another mechanism that may cause esophageal damage is linked to gastroesophageal reflux disease.^15^ When the pH value is higher than 3.5, alendronate is present as a monosodium salt. However, when the pH is below 2, it is mainly present in the form of free acid, which is more irritating to the mucosa.^18^ Therefore, especially in patients who did not remain upright for 30 min after taking the tablet, sodium alendronate, dissolved by stomach acid, may cause specific toxicity to the distal esophagus through reflux.^19^ This was confirmed by another study on animals about the mechanism of alendronate‐induced esophagitis, the process by which alendronate irritates the esophagus is due to its reflux in an acidic state into the esophagus. This irritation is worsened when the patient sleeps down or already has gastroesophageal reflux disease (GERD). Alendronate sodium undergoes conversion to the more irritating free acid at low pH levels, leading to significant esophageal injury, particularly in the presence of reflux.^20^ In recent years, some scholars have also proposed that the pathogenesis may be related to T‐cell‐mediated delayed hypersensitivity reactions, but further studies are needed to confirm it.^15^ Alendronate‐induced pill esophagitis usually has a unique endoscopic manifestation, with segmental extensive erosions, ulcers, and a loose adhesion pseudo‐membrane covered with dense inflammatory exudates.^21^ As can be seen from the picture of this case (Fig. 1a–e), the esophageal segmental mucosa is extensively red and eroded, with ulcer formation of different shades and sizes. These ulcers are circumferentially distributed along the esophageal wall, sometimes in a circular pattern or kissing against each other, with some partial ulcer fusion. The ulcer boundaries are clear, and the overall changes form a patchy pattern. The surface is covered with a prominent, dense, and thick white pseudomembranous exudate that is loosely adherent (Fig. 1c,d). Normal mucosa exists at both ends of the lesion mucosa of the esophagus, stomach, and duodenum, clearly demarcated (Fig. 1a,b). Small patches of normal mucosa and varying degrees of mucosal damage were seen in the lesion segments, further confirming that drug exposure was the main pathogenic mechanism of the disease. Esophageal injury is usually located at the middle and lower end of the esophagus, starting from the second physiological narrow food retention of the esophagus, which is related to the pathogenesis mentioned above. The endoscopic manifestations of alendronate‐induced pill esophagitis exudate are significantly different from those of reflux esophagitis, and esophagitis caused by GERD can be excluded and ruled out by the distal esophagus with intact mucosa.^21^, ^22^

The pathological features of alendronate‐induced pill esophagitis have been reported as nonspecific inflammatory changes, granulation tissue, and fibrinous inflammatory exudate. Enlarged and deeply pigmented multinucleated squamous epithelial giant cells near the lesion site can be seen under microscopy.^18^, ^23^ Refractive crystalline material is present in some biopsy specimens. A detailed history and upper GI endoscopy are key to the diagnosis of this disease. A histopathological biopsy can confirm the nonspecific inflammatory changes and the presence of crystal‐like substances in the esophagus, as well as exclude viral, fungal, and bacterial infections, all of which help confirm the diagnosis of alendronate‐related esophagitis. The pathological examination results of this patient confirmed the presence of inflammatory necrotic tissue with granulation tissue hyperplasia, some squamous epithelium on the surface, and widening of the intercellular space with neutrophil and lymphocyte infiltration, which support the diagnosis of alendronate‐induced pill esophagitis (Fig. 1f–h).

The treatment for alendronate‐induced pill involved immediately discontinuing alendronate sodium and adding a proton pump inhibitor (PPI), mucosal protectants, and other supportive measures after the diagnosis. Most patients can be cured within 1 month after discontinuing the drug, but long‐term use may induce varying degrees of esophageal stricture.^4^, ^24^, ^25^ The risk factors for esophagitis include advanced age, underlying digestive disorders, poor oral hygiene, and difficulty in staying upright after alendronate consumption.^26^ To reduce the possible risk of esophageal injury, patients with achalasia and other esophageal motility disorders, esophageal strictures, or pre‐existing severe reflux esophagitis should avoid taking bisphosphonates.^11^ At the same time, patients taking NSAIDs should use oral drugs of the bisphosphonate group with caution. There are studies that have shown that the two drugs have a synergistic effect on ulcer formation.^27^ To minimize esophageal injury, we recommend that patients should take bisphosphonate tablets half an hour before breakfast, with at least 200–240 mL of water, and maintain an upright position for at least 30 min after swallowing the tablets, or a new formulation of alendronate sodium hydrate (oral jelly) is advised in terms of osteoporosis treatment.^5^, ^6^, ^28^, ^29^

## Conclusion

In short, alendronate‐induced drug esophagitis focuses on prevention and timely diagnosis, and the typical endoscopic manifestations and pathological features of this case can strengthen the understanding of the disease among clinicians and endoscopists and reduce missed diagnosis and misdiagnosis. More research is needed to understand why alendronate can cause esophagus problems and find better ways to treat and prevent them.

### Informed consent

The patient's informed consent was received before the treatment.
